# Polymer Brushes Govern Defect‐Selective Oxidative Etching of Penta‐Twinned Gold Nanorods

**DOI:** 10.1002/anie.8508304

**Published:** 2026-07-29

**Authors:** Tianyi Wu, Irina Koriakina, Feng Li, Zhi‐bo Yang, Yan Zhang, Zitao Chen, Da Wang, Zhihong Nie, Kun Liu, Eugenia Kumacheva

**Affiliations:** ^1^ Department of Chemistry University of Toronto Toronto Ontario Canada; ^2^ Department of Mechanical and Industrial Engineering University of Toronto Toronto Ontario Canada; ^3^ State Key Laboratory of Supramolecular Structure and Materials, College of Chemistry Jilin University Changchun P. R. China; ^4^ State Key Laboratory of Molecular Engineering of Polymers Department of Macromolecular Science Fudan University Shanghai P. R. China; ^5^ Guangdong Provincial Key Laboratory of Optical Information Materials and Technology South China Academy of Advanced Optoelectronics Institute of Electronic Paper Displays South China Normal University Guangzhou P. R. China; ^6^ Department of Chemical Engineering and Applied Chemistry University of Toronto Toronto Ontario Canada; ^7^ Institute of Biomaterials and Biomedical Engineering University of Toronto Toronto Ontario Canada

**Keywords:** crystallographic defects, gold nanorods, oxidative etching, polymer brushes

## Abstract

Oxidative etching of plasmonic nanoparticles provides an effective approach to the variation of their morphology and optical properties, yet polymer ligands are generally treated as passive steric barriers. Here, we show that polymer molecules end‐grafted to the surface of penta‐twinned gold nanorods (AuNRs) perform as active interfacial regulators controlling defect‐selective oxidative metal etching. Through a combination of experiments and simulations, we show that polymer brushes localize etchant ions on the AuNR surface, lowering the energetic barrier for Au atom removal at defect‐rich regions. This defect–ligand interplay leads to the emergence of surface corrugations along the AuNR longitudinal facets, in stark contrast to the etching behavior of single‐crystal AuNR counterparts. By varying polymer molecular weight and grafting density, we achieve precise control of the plasmonic properties of etched AuNRs. These findings establish polymer brushes as active modulators of nanoscale interfacial reaction pathways, providing a strategy for defect‐directed nanoengineering with applications in catalysis, sensing, and photonics.

## Graphical abstract

Polymer brushes act as active interfacial regulators of oxidative etching, directing it to crystallographic defects. In penta‐twinned gold nanorods, defect–ligand interplay localizes etching to defect regions and induces surface corrugations on the long nanorod sides. This work establishes a framework for programmable nanoengineering of the surfaces of metal nanoparticles.

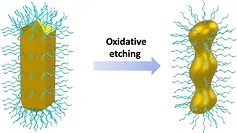

## 1 Introduction

Post‐synthetic oxidative etching of plasmonic nanoparticles (NPs) is a powerful nanofabrication strategy [1, 2, 3]. Instead of a discrete control of NP properties achieved in individual chemical reactions, etching allows for gradual variation in plasmon resonances by changing NP size and shape, without the need for new synthesis. The transfer of zero‐valency surface atoms of NPs into a soluble ionic form has been used for the transformation of gold nanocubes into nanospheres [4], octahedral‐shaped silver nanocrystals into branched octopod structures [5], and palladium nanocubes into hollow nanocages [6], as well as the reduction in dimensions of gold nanorods (AuNRs) [7, 8, 9].

Plasmonic properties are important for AuNR applications in photothermal therapy, where deeper tissue penetration is achieved by utilizing AuNRs with longitudinal surface plasmon resonance (LSPR) centered in the near‐infrared region [10, 11, 12]. In photovoltaic and solar thermal devices for light energy harvesting, the use of AuNRs with the LSPR spanning the visible to near‐infrared spectral range maximizes light absorption and enhances light‐to‐electric energy conversion [13, 14]. Control of the LSPR wavelength, λ_LSPR_, is generally achieved by varying the AuNR length‐to‐diameter ratio (aspect ratio) [15, 16, 17]; however, despite significant progress in conventional [18, 19, 20] and photochemical [21, 22, 23] AuNR syntheses, achieving the exact λ_LSPR_ requires the synthesis of multiple batches.

Oxidative etching, the counterpart of AuNR growth, offers an alternative approach to λ_LSPR_ control, with ligands stabilizing a particular facet against etching. For example, preferential adsorption of hexadecyltrimethylammonium bromide (CTAB) to the {100} or {110} facets of the AuNR long sides led to preferential etching of the tips and the resulting blueshift of λ_LSPR_ [8]. Upon the addition of cysteine preferentially adsorbing at the CTAB‐capped AuNR tips, the etching process was directed towards the long AuNR sides and resulted in the corresponding redshift in λ_LSPR_ [8].

Oxidative etching of polymer‐coated metal NPs has been reported [8, 24, 25], however, polymers have been largely treated as passive steric barriers controlling diffusion‐driven etchant transport to the NP surface. Polymer chemisorption to the metal surface can fundamentally alter local chemical environments and potentially influence the spatial (site‐specific) reactivity, particularly in nanostructures containing crystallographic defects. This distinction is especially relevant for penta‐twinned AuNRs, which possess defect‐rich twin boundaries, edge dislocations, and stacking faults that exhibit distinct energetic and chemical characteristics [26, 27]. Yet, the question of whether polymer ligands can actively regulate defect reactivity and thereby direct etching pathways remains largely unexplored. Beyond acting as steric barriers, polymer molecules attached to the metal surface in a brush‐like configuration introduce a confined, chemically active interfacial environment where reactant localization, ligand coordination, and surface‐atom activation are intrinsically coupled. In such environments, oxidative etching can deviate from classical diffusion‐limited behavior, particularly for nanostructures containing crystallographic defects with distinct local energetics. The ability of polymer brushes to selectively modulate defect reactivity and thereby direct etching pathways represents an unexplored research direction.

Here, we report a combined experimental and theoretical study of oxidative etching of penta‐twinned AuNRs capped with end‐grafted polymer in a brush‐like configuration. We show that polymer brushes regulate the transport of etchant species and actively modulate the reactivity of crystallographic defects, lowering the energy barrier for Au atom removal at defect‐rich regions. This defect–ligand interplay leads to the emergence of surface corrugation along the longitudinal facets of AuNRs, in stark contrast to the morphology of single‐crystal AuNR counterparts etched under the same conditions. By systematically varying polymer molecular weight and grafting density, we establish a framework for programming defect‐selective etching of AuNRs and controlling their plasmonic properties. These findings position polymer brushes as active regulators of nanoscale reaction pathways and provide a strategy for defect‐directed engineering of plasmonic nanostructures.

## 2 Results and Discussion

Penta‐twinned and single‐crystal AuNRs (used as control) were synthesized using the protocols reported elsewhere [28, 29] and subsequently separated from byproduct spherical NPs using depletion flocculation [30, 31] (Supporting Information). The AuNRs were subjected to ligand exchange, in which the hexadecyltrimethylammonium chloride ligands were replaced with thiol group‐terminated polystyrene (PS) molecules with number‐average molecular weights of 5 300, 25 000, or 50 000 g/mol, corresponding to degrees of polymerization of 51, 240, and 480 and denoted as PS‐5K, PS‐25K, and PS‐50K, respectively. Ligand exchange was performed in *N,N*‐dimethylformamide (DMF), a good solvent for PS (the second virial coefficient, *A*_2_ = 3.5 × 10^−^^4^ mol cm^3^ g^−^^2^; Flory–Huggins interaction parameter, *χ* = 0.46) [32]. The polymer grafting density, σ, on the surface of penta‐twinned AuNRs was determined using an NMR‐based method [33] (Figures S1 and S2). The value of σ was controlled by varying the ratio between the polymer mass concentration and the AuNR surface in the ligand exchange process. Etching of the AuNRs was performed in DMF using FeCl_3_ at the molar ratio *R* = [Fe^3^^+^]/[Au^0^], where 2.0 ≤ *R* ≤ 20.0.

**FIGURE 1:**
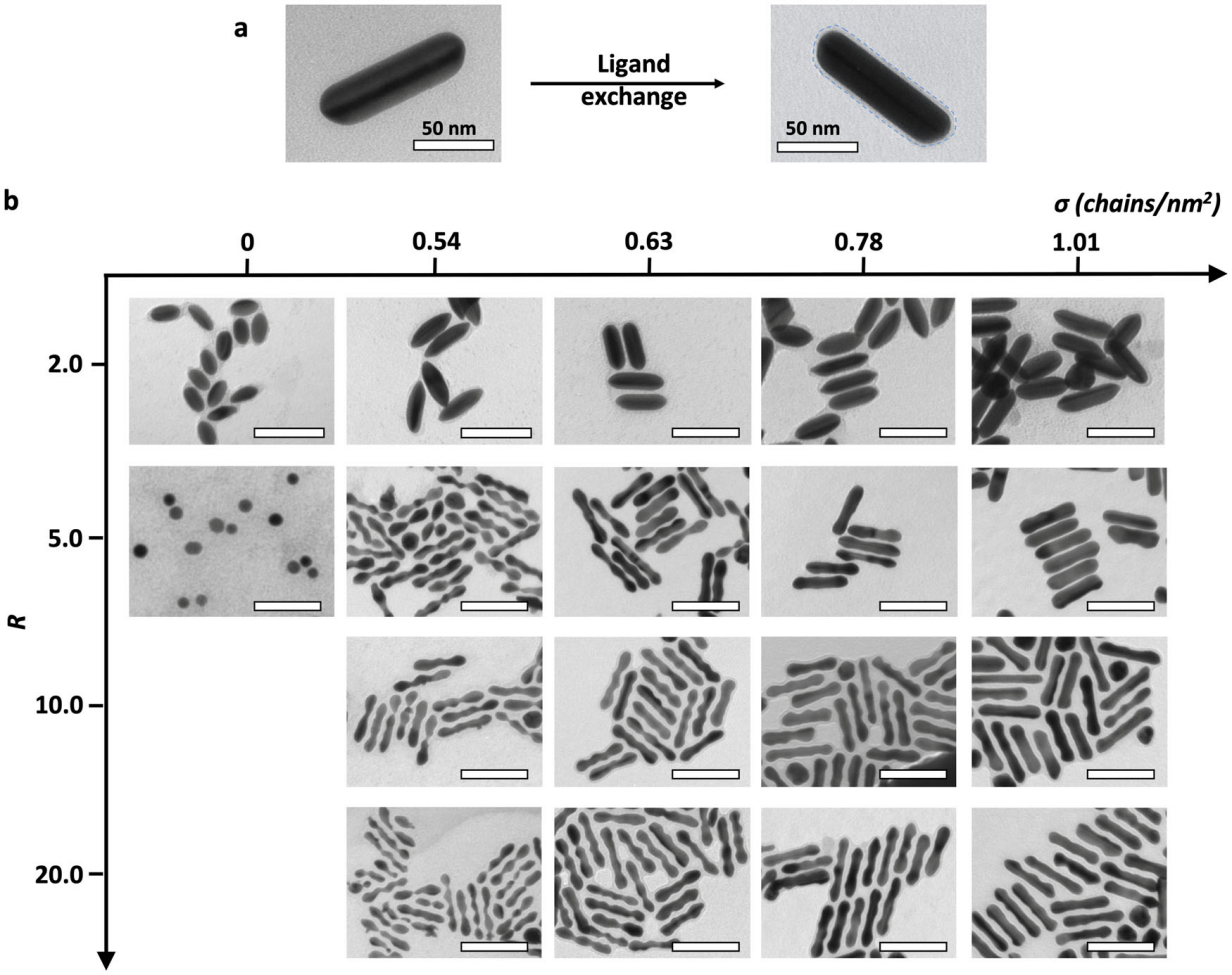
TEM images of penta‐twinned AuNRs subjected to oxidative etching. (a) Pristine AuNRs prior to (left) and following ligand exchange with PS‐5K to σ = 0.78 ± 0.05 chains/nm^2^ (right). The dashed line profiles the polymer layer for visualization purposes. (b) AuNRs capped with PS‐5K at varying σ and subjected to oxidative etching at varying *R* for 15 h. Scale bars are 100 nm.

Figure 1a shows representative transmission electron microscopy (TEM) images of an individual penta‐twinned AuNR prior to (left) and following ligand exchange with PS‐5K (right). The average AuNR length and diameter were 92 ± 7 and 27 ± 5 nm, respectively, with ligand exchange resulting in a 2.4 ± 0.3 nm‐thick polymer shell on the surface of dried AuNRs. Figure 1b shows representative TEM images of the AuNRs subjected to 15 h‐long etching. At *R* = 2.0, the AuNRs capped with hexadecyltrimethylammonium ligands gained a rice‐like shape and retained a smooth surface, and at *R* = 5.0, they transformed into spherical NPs. Further increase in *R* resulted in complete AuNR dissolution. Polymer‐capped AuNRs acquired a rice‐like shape at *R* = 2.0; at 5.0 ≤ *R* ≤ 20.0, they retained a nanorod appearance and gained surface pits on the long side, similar to etched polymer‐coated silver nanowires [34] and palladium nanorods [35]. At σ = const., with increasing *R*, the AuNR morphology changed from mild roughening to distinct pitting. Surface corrugations were less prominent with σ > 0.78 chains/nm^2^, while at σ = 0.22 ± 0.05 chains/nm^2^, significant AuNR defragmentation was observed (Figure S3). The statistical analysis of the corrugated AuNRs was performed for their 2D projections in the TEM images. To mitigate the limitations of 2D imaging, we analyzed a large dataset of the AuNRs randomly oriented on the TEM grid (*n* > 200 per etching condition). In addition, a 3D AuNR reconstruction (Movie S1) was performed, which confirmed that the corrugation occurs throughout the entire surface of the AuNR long side.

Notably, single‐crystal AuNRs capped with PS‐5K at σ = 0.29 ± 0.11 chains/nm^2^ and subjected to 15 h etching at *R* = 5.0 retained a smooth surface (Figure S4) and exhibited an insignificant change in dimensions, with average AuNR length decreasing from 52 ± 6 to 47 ± 6 nm and the average diameter remaining at 17 ± 3 nm. Different etching patterns of single‐crystal and penta‐twinned AuNRs were attributed to their crystallographic structures, as discussed below.

**FIGURE 2:**
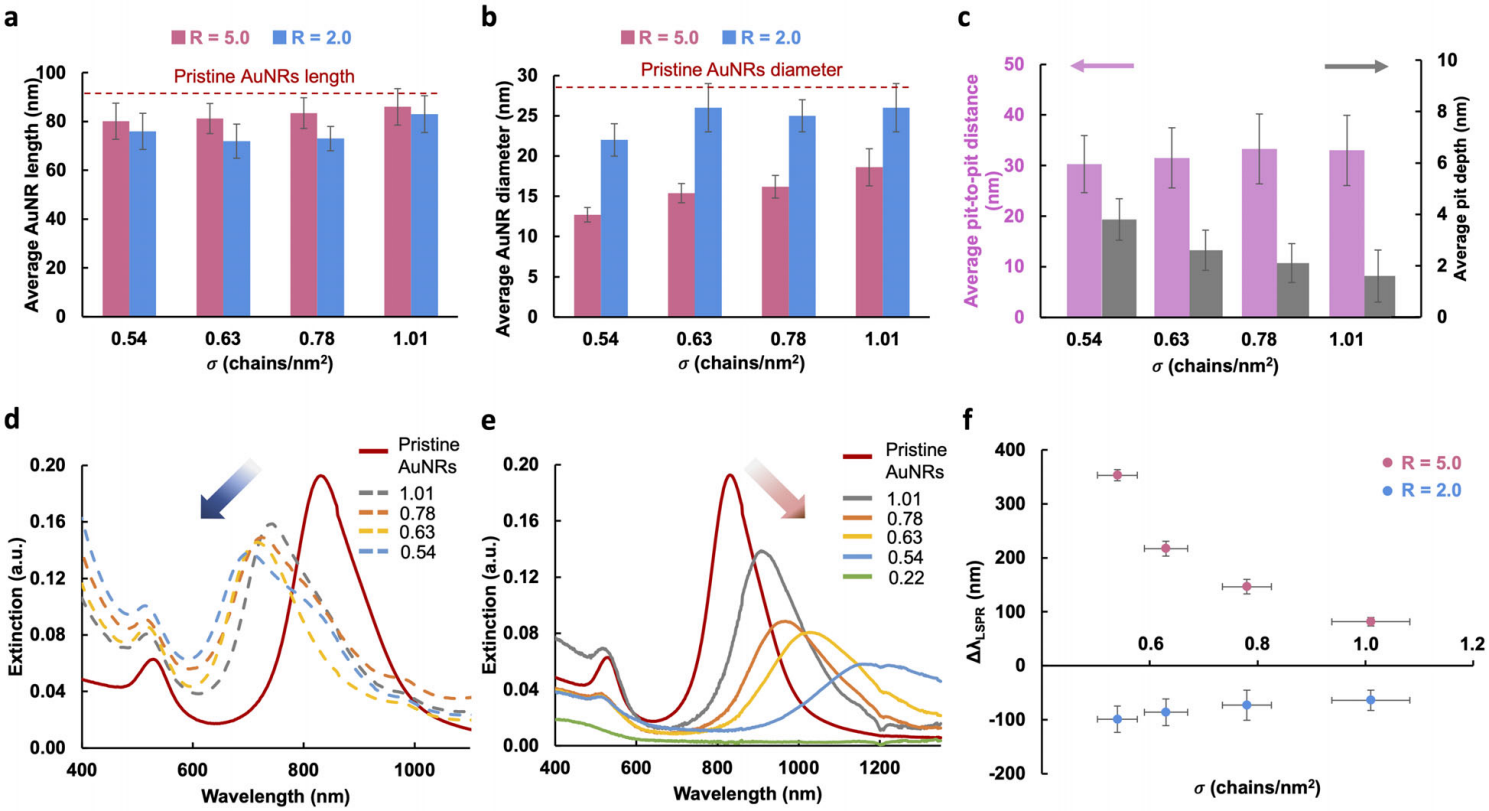
Properties of etched AuNRs. (a) Variation in the average length of AuNRs, plotted versus σ at *R* = 2.0 (blue) and *R* = 5.0 (red). (b) Variation in the average diameter of AuNRs capped with PS‐5K at varying σ for *R* = 2.0 (blue) and *R* = 5.0 (red). The length and diameter of the AuNRs prior to etching are shown with dashed horizontal lines in (a) and (b). (c) Average pit‐to‐pit distance (purple) and pit depth (gray) for PS‐5K‐coated AuNRs etched at *R* = 5.0. (d) Evolution of spectra of polymer‐functionalized AuNRs at varying σ and subjected to etching at *R* = 2.0 (dashed lines). (e) Evolution of spectra of polymer‐functionalized AuNRs at varying σ and etched at *R* = 5.0 (solid lines). In (d) and (e), the spectrum of AuNRs prior to etching is shown with solid red lines, and the values of σ are indicated in the legend. (f) Etching‐induced changes in Δλ_LSPR_ plotted versus σ for *R* = 2.0 (blue symbols) and *R* = 5.0 (red symbols). The results presented in (a–c) were obtained using automated analysis of TEM images for > 200 AuNRs per etching condition. The error bars represent the standard deviation from three independent experiments. The AuNRs were capped with PS‐5K, and the etching time was 15 h.

Figure 2a–c shows the results of the analysis of TEM images of etched AuNRs coated with PS‐5K at varying σ. At *R* = 2.0 and 5.0, the average AuNR length decreased, with a stronger reduction at *R* = 2.0 (Figure 2a). No statistically significant dependence of the AuNR length on σ was observed for either value of *R*. The corresponding changes in the average effective AuNR diameter are shown in Figure 2b. The details of the calculation of AuNR diameter are provided in Figure S5. At both *R* ratios, the average AuNR diameter decreased in comparison with pristine AuNRs, with a stronger reduction at *R* = 5.0 due to the formation of surface pits. Under these latter conditions, denser polymer grafting provided greater AuNR protection against etching. Figure 2c shows the average pit‐to‐pit distance and pit depth for the AuNRs etched at *R* = 5.0. The illustration of the corresponding AuNR morphology is shown in Figure S5. With increasing σ, the average pit‐to‐pit distance remained constant, while the average pit depth reduced.

Oxidative etching of AuNRs resulted in changes in their ultraviolet–visible–near‐infrared (UV–Vis–NIR) spectra (Figure 2d,e). At *R* = 2.0, with a stronger decrease in the AuNR length and a slight decrease in the diameter, the resulting AuNR aspect ratio led to a blueshift in λ_LSPR_, with a stronger trend observed at lower σ. In contrast, at *R* = 5.0, the change in the AuNR length was weak, while their effective diameter decreased more substantially, resulting in an increase in aspect ratio and a significant redshift in λ_LSPR_ (Figure 2e). A stronger redshift occurred at lower σ, accompanied by plasmonic peak broadening. Similar trends were observed at *R* of 10.0 and 20.0 (Figure S6). Markedly, under all etching conditions, extinction intensity reduced due to the reduction in the AuNR mass in solution. Figure 2f summarizes the changes in the spectral position of λ_LSPR,_ plotted as a function of σ. At *R* = 5.0, λ_LSPR_ underwent redshift (Δλ_LSPR_ > 0), while at *R* = 2.0 a blueshift in λ_LSPR_ was observed, with Δλ_LSPR_ < 0. The etched AuNRs were colloidally stable during the etching process and after 90‐day storage in the dry state, following their redispersion in DMF (Figure S7).

**FIGURE 3:**
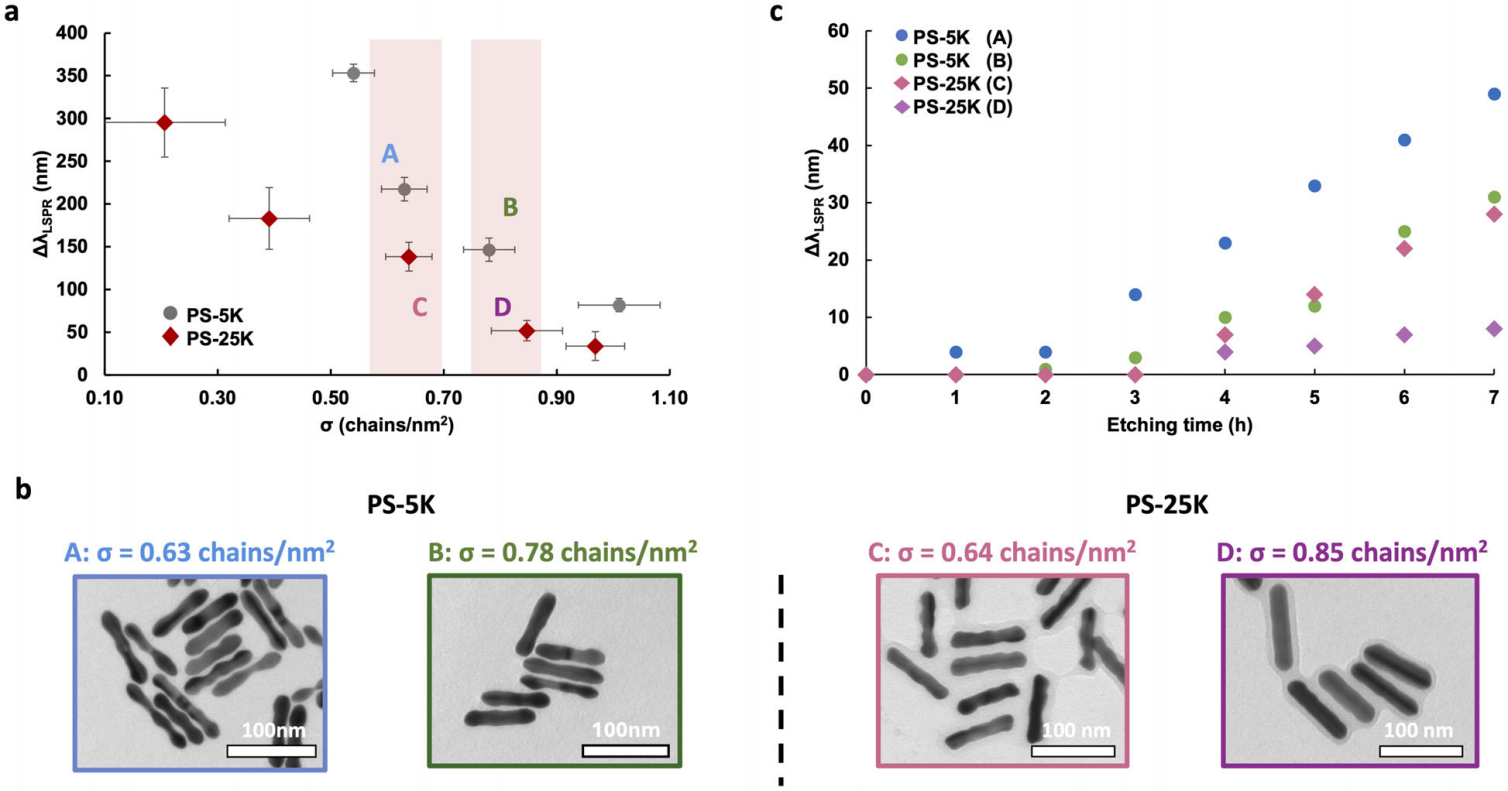
Effect of the molecular weight of polymer ligands on AuNR etching. (a) Variation in λ_LSPR_ for the etched AuNRs capped with PS‐5K or PS‐25K at varying σ. The horizontal and vertical error bars represent the standard deviations of the polymer grafting density and the Δλ_LSPR_, respectively, derived from three independent experiments. The AuNRs were etched at *R* = 5.0 for 15 h. (b) Representative TEM images of AuNRs coated with PS‐5K and PS‐25K at comparable σ after 15 h of etching. (c) Etching kinetics for AuNRs coated with PS‐5K and PS‐25K. In (a–c), *R* = 5.0 was used.

To explore the effect of polymer ligands on AuNR etching, we examined the changes in the λ_LSPR_ spectral position of the AuNRs capped with PS‐5K and PS‐25K with varying σ (Figure 3). Figure 3a shows that, at both low and high σ (marked as points A and B, respectively), etched AuNRs tethered with PS‐5K exhibited a stronger shift in λ_LSPR_, compared to the AuNRs coated with PS‐25K at similar low and high σ (marked as points C and D, respectively). The effect of polymer molecular weight on the λ_LSPR_ shift was more significant at a lower σ. These results correlated with changes in the morphology of etched polymer‐capped AuNRs. The corresponding TEM images in Figure 3b show that, at a similar σ, PS‐5K–functionalized AuNRs exhibited stronger surface corrugation than those capped with PS‐25K. The TEM images of etched AuNRs capped with PS‐25K and the corresponding statistical analyses of their structural characteristics are given in Figure S8. Consistent with published reports on non‐etched (smooth surface) AuNRs [15, 29, 36], a higher aspect ratio resulted in a more significant change in Δλ_LSPR_ (Figure S9). We note that for PS‐50K the maximum σ achieved was only 0.07 ± 0.02 chains/nm^2^, and under the same etching conditions the corresponding AuNRs transformed into nanospheres (Figure S10).

Figure 3c reveals the etching kinetics for the AuNRs capped with PS‐5K or PS‐25K at σ designated with points A‐D in Figure 3a. An incubation period of 1 and 3 h with no changes occurred in λ_LSPR_ was observed for the AuNRs tethered with PS‐5K and PS‐25K, respectively. Beyond this time period, a greater and faster change in λ_LSPR_ was favored at a lower σ for the AuNRs tethered with a lower molecular weight polymer.

To decouple the role of the polymer chain from that of the thiol group, control experiments were performed by etching AuNRs capped with 1‐dodecanethiol (DDT). At *R* = 10.0 and 15 h etching (similar to the conditions used for AuNRs end‐capped with PS‐5K), the DDT‐functionalized AuNRs transformed into smooth‐surface rice‐shaped NPs (Figure S11). This result was in striking difference with the formation of surface corrugations on the surface of AuNRs end‐capped with PS‐5K or PS‐25K.

The results shown in Figure 3 signify a superposition of the effect of polymer molecular weight and σ on the AuNR etching, that is, a similar Δλ_LSPR_ was achieved for a particular combination of the higher σ and a lower polymer molecular weight (marked with point B), and the lower σ with a higher polymer molecular weight (marked with point C). To evaluate the role of polymer characteristics, we estimated the thickness of the polymer layer. Based on the range of σ used, the intermolecular spacing *d* (= σ^−1/2^ ) of the polymer molecules on the AuNR surface was in the range of 1.1–1.3 nm for both PS‐5K and PS‐25K. For these polymers, we used the Flory mean‐field approach [37] and estimated the thickness of the polymer brush layer to be 2.7–2.9 nm (PS‐5K) and 13.0–14.3 nm (PS‐25K) (see details of the calculations in Table S1). At a similar grafting density σ corresponding to point A and point C in Figure 3, the polymer layer formed by PS‐25K was approximately five‐fold thicker than that formed by PS‐5K. Furthermore, the energy‐dispersive x‐ray (EDX) elemental mapping (Figure S12) confirmed the presence of Fe species on the etched AuNR surface. We note that previous studies have reported weak coordination between Fe^3^^+^ ions and the electron‐rich aromatic rings of PS via cation–*π* interactions [38, 39].

**FIGURE 4:**
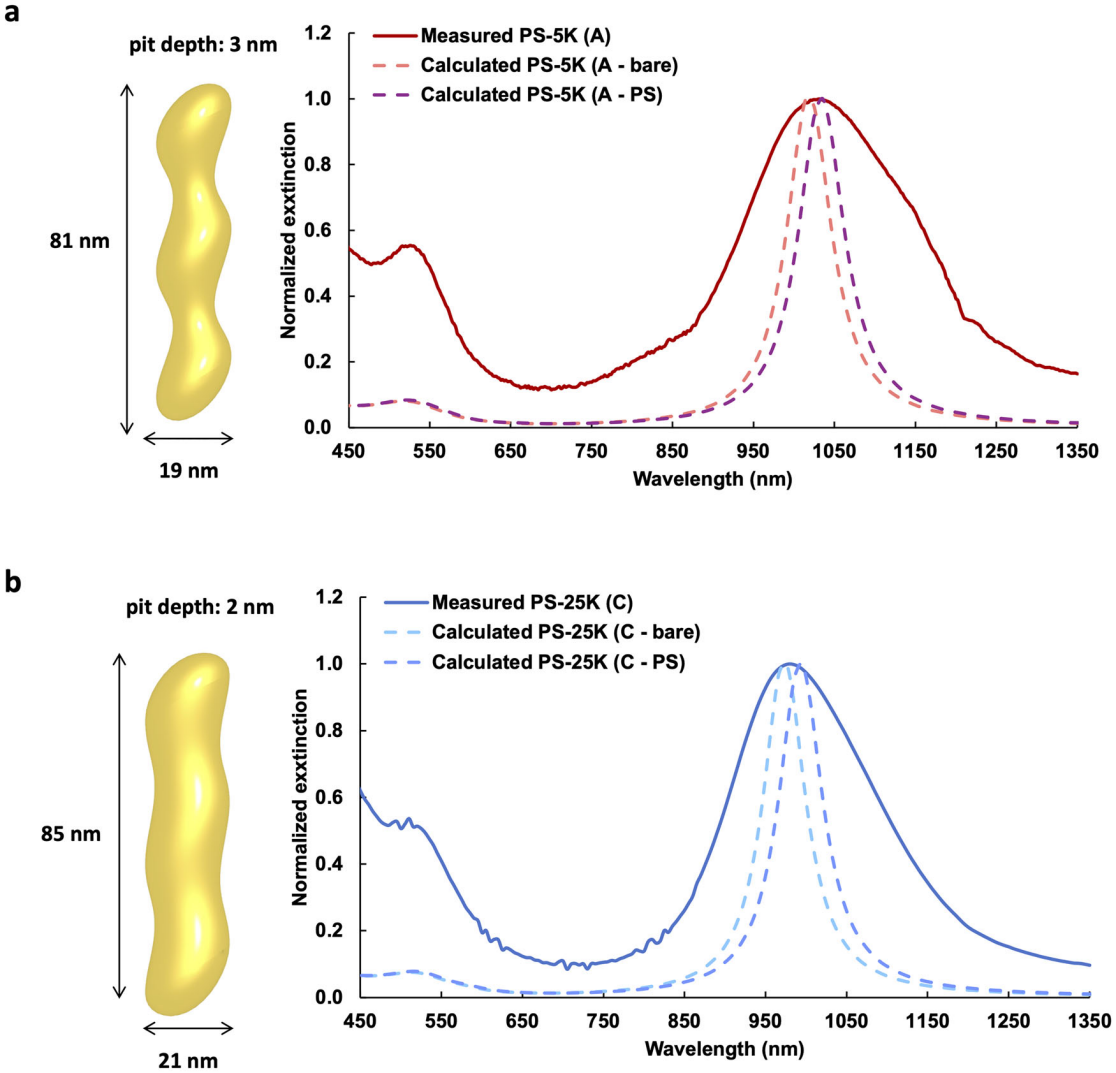
FDTD simulation of spectra of etched AuNRs. (a) *Left*: A AuNR capped with PS‐5K (corresponding to condition A in (a)). *Right*: Experimentally acquired (solid red line) and a simulated extinction spectra of bare AuNRs (dashed orange line) and the AuNRs carrying a swollen PS layer (dashed purple line); (b) *Left*: A AuNR capped with PS‐25K (corresponding to condition C in (a)). *Right*: Experimentally acquired (solid blue line) and a simulated extinction spectra of bare AuNRs (dashed light blue line) and the AuNRs coated with a swollen PS layer (dashed dark blue line).

To validate the impact of etching on AuNR plasmonic properties, we performed finite‐difference time‐domain (FDTD) simulations of their extinction spectra by numerically solving Maxwell's equations (Figure 4). The simulated AuNR geometries corresponded to those obtained under etching conditions A and C (Figure 3a), with average dimensions extracted by analyzing their TEM images. The simulations were performed for the ligand‐free AuNRs (denoted as PS‐5K (A‐bare) or PS‐25K (C‐bare)) and for the AuNRs carrying a swollen polymer brush layer (denoted as PS‐5K (A‐PS) or PS‐25K (C‐PS)). The details of the simulation parameters are provided in Supporting Information. Due to the higher refractive index of the PS layer than that of the surrounding DMF solvent, the simulated spectra of the PS‐coated AuNRs exhibited a redshift in λ_LSPR_, compared to their bare counterparts. The simulated spectral positions of λ_LSPR_ agreed with the experimentally measured difference in λ_LSPR_ for etched AuNRs (points A and C, respectively). The difference originated from deeper surface pits induced by etching under condition A, that is, a greater effective average AuNR aspect ratio. The experimentally acquired spectra exhibited broader plasmonic bands, consistent with moderate polydispersity in AuNR dimensions.

Since the results of the etching of single‐crystal and penta‐twinned AuNRs showed a striking difference, we focused on the structure of the latter AuNRs and performed atomic‐scale density functional theory (DFT) simulations. Penta‐twinned AuNRs contain {111} facets at the tips and {100} facets at the longitudinal side faces [15]. In addition, these AuNRs contain defect‐rich regions, such as twin boundaries, edge dislocations, and stacking faults at facet intersections [13, 26, 40]. For the defect‐free (pristine) and defect‐containing regions of these facets, we used two energetic descriptors, that is, (i) the chemisorption‐driven binding energy, *E*_b_, of the thiol group, and (ii) the energy of Au─Au vacancy formation, *E*_v_, in the presence of the surface‐attached thiol group (Table 1). Consistent with prior theoretical works [41, 42], we used surface vacancy motifs for atomistic representation of crystallographic defects, thus capturing the essential features of local atomic under‐coordination in defect regions (termed as vacancies). To reduce the computational complexity while isolating the intrinsic Au–S coordination chemistry, a single styrene–thiolate unit was used as a minimal molecular model to represent thiol group binding, thereby capturing covalent Au–S interactions and neglecting long‐range steric and entropic contributions from the entire polymer molecule. The details of DFT simulations are provided in Supporting Information.

**TABLE 1:** DFT‐calculated energy descriptors for the {100} facet of AuNR.

Surface region of {100} facet	*E*_b_ (eV)	*E*_v_ (eV)
Pristine region	−1.69	1.09
Vacancy region	−1.78	0.91

As shown in Table 1, adsorption of the thiol group was energetically favorable on both pristine (defect‐free) and vacancy‐containing {100} facets, however, the binding energy, *E*_b_, was slightly lower at the defect regions (*E*_b_ =  −1.78 eV), indicating a weak preference for thiol group coordination to defects, compared to pristine regions (*E*_b_ = −1.69 eV). This result was consistent with earlier reports on the preferred adsorption of thiol molecules on defect regions of noble metal surfaces, caused by enhanced local reactivity associated with atomic under‐coordination [43, 44]. More importantly, a stronger reduction in the energy of formation of Au─Au vacancy, *E*_v_, for defect regions (*E*_v_ = 0.91 eV), relative to the defect‐free location (*E*_v_ = 1.09 eV) indicated that Au─Au bond cleavage was more favorable at thiol group‐bound defect sites. Comparison of *E*_v_ and *E*_b_ values for pristine and defective locations of {100} facets revealed the competition between the thiolate‐mediated surface passivation and defect‐facilitated Au atom removal, where a markedly lower *E*_v_ versus *E*_b_ promoted preferential etching at crystallographic defects.

**FIGURE 5:**
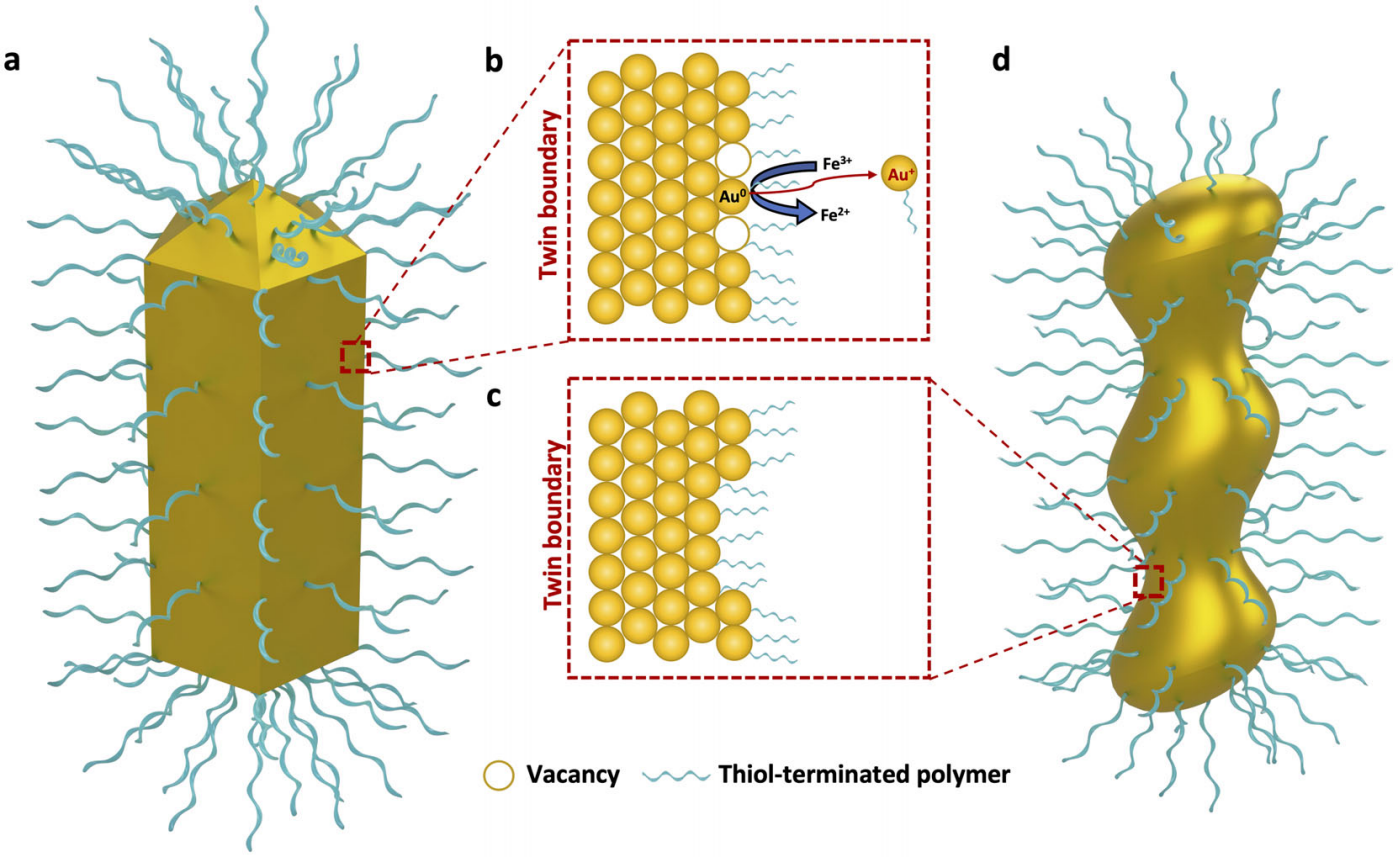
Proposed pathway of oxidative etching of polymer‐capped penta‐twinned AuNRs. (a) Polymer end‐tethered AuNR prior to etching. (b) Atomic‐scale schematic of a defect‐rich region (twin boundary) during the etching process. (c) Atomic‐scale schematic of a defect‐rich region (twin boundary) after etching, illustrating polymer migration. (d) Etched AuNRs coated with end‐tethered polymer.

Figure 5 illustrates the proposed pathway of the oxidative etching of polymer‐capped penta‐twinned AuNRs and the subsequent formation of surface corrugations on the long AuNR sides. Due to a 7.35° angle misfit inherent to the penta‐twinned geometry, these AuNRs possess defect‐rich twin boundaries along their longitudinal sides. Prior to etching, thiol group‐terminated polymer molecules were grafted to the AuNR surface (Figure 5a) with weak preferential chemisorption of thiolates to defect regions, driven by the lower *E*_b_ (Table 1). Upon exposure to Fe^3^^+^ ions, etching resulted in the oxidation of the surface atoms from Au^0^ to the Au^+^ state (Figure 5b) [1, 7]. The latter species could coordinate with surface‐bound thiolates, thus forming transient complexes that facilitated Au atom removal from the AuNR surface [33, 45]. Due to a markedly lower *E*_v_ (Table 1), the defect regions along the longitudinal {100} facets of the AuNRs' long sides underwent preferential etching. This site‐selective process is consistent with etching of surfaces capped with small‐molecule thiols, in which etching is localized to sites where ligands are initially lost and cannot be quickly replaced [46, 47, 48]. While the PS chains are temporarily outpaced during the active etching event, the continuous lateral mobility of gold‐thiolate species allows polymer chains to be redistributed toward the newly exposed under‐coordinated Au atoms (Figure 5c) [4, 43, 44]. As a result, the entire surface of AuNRs, including etched pits, was coated with a polymer shell (Figures 5d and 1c). This observation, along with the colloidal stability of etched AuNRs in DMF, supported polymer retention on their surface.

Preferential etching of the AuNR tips at low etchant concentrations (*R* = 2.0) was attributed to the splaying of end‐grafted polymer chains radially outward, due to the higher curvature of the tips [49, 50, 51], which favored the exposure of the AuNR surface to Fe^3+^ ions. At higher *R*, more etchant ions reached the surface of {100} facets of the long AuNR sides, resulting in the etching of the defect regions.

The role of the polymer ligands in the etching process can be understood in terms of the polymer molecular weight and σ, which determines the thickness and the extent of packing of the PS layer acting as a steric barrier for the diffusion of Fe ^3+^ ions to the AuNR surface. At high PS molecular weight or high σ, the AuNR surface was passivated against oxidative etching, while at low σ the AuNRs disintegrated or transformed into spherical NPs and ultimately dissolved. The dependence of etching kinetics on polymer molecular weight and σ was consistent with diffusion‐limited ion transport imposed by the polymer layer thickness.

Unlike single‐crystal AuNRs, whose crystal lattice is continuous throughout the long side of the particle, penta‐twinned AuNRs exhibit defects due to the intrinsic geometric 7.35° mismatch gap of five‐fold cyclic twinning [26]. The mismatch results in lattice distortion, crystallographic symmetry reduction, and the formation of strain‐relief defects, such as axial dislocations and stacking faults [26, 52]. Thus, penta‐twinned AuNRs exhibit a more complex defect structure and strain distribution than those of single‐crystal AuNRs. Interestingly, Liz‐Marzán et al. have recently reported a major difference in the handedness of chiral AuNRs obtained by the overgrowth of gold on the surface of single‐crystal and penta‐twinned seed AuNRs by using the same chiral inducer enantiomer [27]. These results reinforce the role of crystallographic structure, which may be pertinent to the difference in both etching and overgrowth of single‐crystal and penta‐twinned AuNRs.

Finally, we note that beyond controlling the spectroscopic properties of AuNRs, polymer‐mediated etching resulted in the emergence of local high‐curvature regions on the AuNR surface and significantly increased the surface area of etched AuNRs, in comparison with pristine NPs, which may provide opportunities for their applications in catalysis [53, 54]. Furthermore, while the present work focused on penta‐twinned AuNRs, other gold nanostructures, for example, decahedral or bipyramidal NPs containing twin‐boundary defects [55, 56], may exhibit a similar defect‐selective polymer‐mediated etching behavior. Extending polymer‐mediated etching strategies to a broader range of multi‐twinned NPs would stimulate further studies in this field of research.

## 3 Conclusion

This work shows that polymer brush‐mediated oxidative etching of AuNRs provides a synthetic handle for controlling their plasmonic properties, with either blueshift or redshift in λ_LSPR_. The location and extent of etching, as well as etching kinetics, were governed by the superposition of polymer molecular weight and grafting density. The mechanistic insights developed in this work may inform the design of shape‐controlled nanostructures for plasmonic and catalytic applications. More broadly, our findings show that end‐grafted polymer ligands do not merely act as passive surface stabilizers but are active regulators of nanoscale reaction pathways. Polymer‐mediated etching thus offers a strategy for controlling complex morphologies of plasmonic NPs.

## Author Contributions

**Tianyi Wu**: methodology, conceptualization, investigation, formal analysis, writing – original draft, writing – review and editing, validation. **Irina Koriakina**: methodology, investigation, validation, formal analysis, writing – original draft, writing – review and editing. **Feng Li**: formal analysis, writing – review and editing. **Zhi‐bo Yang**: formal analysis, writing – review and editing. **Yan Zhang**: writing – review and editing, formal analysis. **Zitao Chen**: writing – review and editing, formal analysis, visualization. **Da Wang**: writing – review and editing, formal analysis, visualization. **Zhihong Nie**: writing – review and editing, formal analysis. **Kun Liu**: writing – review and editing, formal analysis. **Eugenia Kumacheva**: conceptualization, methodology, supervision, resources, project administration, funding acquisition, writing – original draft, writing – review and editing, validation, formal analysis, investigation.

## Conflicts of Interest

The authors declare no conflicts of interest.

## Supporting information



The authors have cited additional references within the Supporting Information [57, 58, 59, 60, 61, 62, 63, 64, 65, 66, 67].**Supporting File 1**: anie73827‐sup‐0001‐SuppMat.docx.

**Supporting File 2**: anie73827‐sup‐0002‐MovieS1.mp4.

## Data Availability

The data that support the findings of this study are available from the corresponding author upon reasonable request.
