# Supplementary material for: Polymer Brushes Govern Defect‐Selective Oxidative Etching of Penta‐Twinned Gold Nanorods
Source: Angew Chem Int Ed Engl. 2026 Jul 29;65(39):e8508304. doi: 10.1002/anie.8508304 (PMC13592317; doi:10.1002/anie.8508304)
Supplement: Supplementary file 1 [file ANIE-65-e8508304-s002.docx]

Supporting Information

**Polymer Brushes Govern Defect-Selective Oxidative Etching Of Penta-Twinned Gold Nanorods**

Tianyi Wu ^a^, Irina Koriakina ^a^, Feng Li ^d^, Zhi-bo Yang ^e^, Yan Zhang ^f^, Zitao Chen ^g^, Da Wang ^g^, Zhihong Nie ^f^, Kun Liu ^e^ and Eugenia Kumacheva*^a, b, c^

[a]	T. Wu, Dr. I. Koriakina, Prof. E. Kumacheva
Department of Chemistry
University of Toronto
Toronto, Ontario, M5S 3H6, Canada
E-mail: eugenia.kumacheva@utoronto.ca

[b]	Prof. E. Kumacheva
Department of Chemical Engineering and Applied Chemistry
University of Toronto
Toronto, Ontario, M5S 3E5, Canada

[c]	Prof. E. Kumacheva
Institute of Biomaterials and Biomedical Engineering
University of Toronto
Toronto, Ontario, M5S 3G9, Canada

[d]	Dr. F. Li
Department of Mechanical and Industrial Engineering
University of Toronto
Toronto, Ontario, M5S 3G8, Canada

[e]	Z-B. Yang, Prof. K. Liu
State Key Laboratory of Supramolecular Structure and Materials, College of Chemistry
Jilin University
Changchun, 130012, P. R. China

[f]	Dr. Y. Zhang, Prof. Z. Nie
State Key Laboratory of Molecular Engineering of Polymers, Department of Macromolecular Science
Fudan University
Shanghai, 200438, P. R. China

[g]	Dr. Z. Chen, Prof. D. Wang
Guangdong Provincial Key Laboratory of Optical Information Materials and Technology, South China Academy of Advanced Optoelectronics, Institute of Electronic Paper Displays
South China Normal University
Guangzhou, 510006, P. R. China

Table of Contents

[1.	Experimental Section	3](#_Toc232800869)

[1.1	Materials	3](#_Toc232800870)

[1.2	Methods	3](#_Toc232800871)

[**1.2.1 Synthesis of penta-twinned gold nanorods (AuNRs).**	3](#_Toc232800872)

[**1.2.2 Synthesis of single-crystal AuNRs.**	4](#_Toc232800873)

[**1.2.3 Depletion-induced fractionation of AuNRs.**	4](#_Toc232800874)

[**1.2.4. Functionalization of AuNRs with thiol-terminated polystyrene.**	4](#_Toc232800875)

[1.3	Characterization of AuNRs	4](#_Toc232800876)

[2.	Etching of penta-twinned AuNRs functionalized with PS-5K	7](#_Toc232800877)

[3.	Etching of single-crystal AuNRs functionalized with PS-5K	7](#_Toc232800878)

[4.	Analysis of the topography of etched penta-twinned AuNRs	8](#_Toc232800879)

[5.	Extinction spectra of etched AuNRs functionalized with PS-5K	9](#_Toc232800880)

[6.	Stability of Etched AuNRs	10](#_Toc232800881)

[7.	Etching of penta-twinned AuNRs functionalized with PS-25K	10](#_Toc232800882)

[8.	Correlation between optical and morphological evolution	11](#_Toc232800883)

[9.	Etching of penta-twinned AuNRs functionalized with PS-50K	12](#_Toc232800884)

[10.	Etching of AuNRs functionalized with alternative thiol-terminated ligands	13](#_Toc232800885)

[11.	Calculation of thickness of the polymer layer on the AuNR surface	14](#_Toc232800886)

[12.	Energy-dispersive X-ray elemental mapping of etched AuNRs	15](#_Toc232800887)

[13.	Simulations	16](#_Toc232800888)

[13.1	Finite-difference time-domain simulations	16](#_Toc232800889)

[13.2	Density functional theory simulations	17](#_Toc232800890)

[References	19](#_Toc232800891)

# 1. **Experimental Section**

## 1.1  **Materials**

Gold(III) chloride solution (HAuCl_4_, 99.99%, 30 wt% in dilute HCl), citric acid (≥ 99.5%), sodium borohydride (NaBH_4_, > 99%), L-ascorbic acid (AA, ≥ 99%), silver nitrate (AgNO_3_, ≥ 99%), hexadecyltrimethylammonium bromide (CTAB, BioXtra, ≥ 99%), hexadecyltrimethylammonium chloride (CTAC, 25 wt% in water), iron(III) chloride (FeCl_3_, 97%), 1,3,5-Trimethoxybenzene (TMB, ReagentPlus, ≥ 99%), Dichloromethane-d_2_ (DCM-d_2_, 99.9 atom % D), *N,N*-dimethylformamide (DMF), and 1-dodecanethiol (DDT, ≥ 98% ), were purchased from Sigma-Aldrich. Thiol-terminated polystyrene (PS) with a number average molecular weight, Mn, of 5 300, 25 000, and 50 000 g/mol, with polydispersity indices of 1.1, 1.07, and 1.01, respectively, (synthesized by anionic polymerization) was purchased from Polymer Source, Inc. All chemicals were used without further purification. Deionized water (resistivity 18.2 MΩ·cm) was obtained from the was sourced from the Milli-Q Reference Water Purification System. Carbon-coated copper grids (300-mesh) were purchased from Electron Microscopy Sciences.

## 1.2  **Methods**

**1.2.1 Synthesis of penta-twinned gold nanorods (AuNRs).** Penta-twinned AuNRs were synthesized following a modified seeded growth procedure reported elsewhere.^[1]^ First, Au seeds were synthesized by combining HAuCl_4_ (166 μL, 15 mM), CTAC solution (5 mL, 0.1 M), and citric acid (500 μL, 0.1 M) in a 20 mL scintillation vial. After stirring at room temperature for 30 min, ice-cold NaBH_4_ (250 μL, 25 mM) was rapidly introduced to this solution under vigorous stirring for 2 min, which resulted in a color change of the mixture from pale yellow to light-brown. The seed solution was then allowed to rest at room temperature for 15 min, which was followed by its gentle stirring at 80 °C in an oil bath for 90 min.

The growth solution was prepared by dissolving CTAB (800 mL, 8 mM) and HAuCl_4_ (768 μL, 0.13 M), under stirring at room temperature. After 15 min, AA (2 mL, 0.1 M) was rapidly added sunder vigorous stirring, causing the color change of the solution from orange to colorless. A volume of 2.4 mL of seed solution, was rapidly injected into the growth solution under vigorous stirring for 1 min. The resulting solution was left undisturbed overnight.

**1.2.2 Synthesis of single-crystal AuNRs.** Single-crystal AuNRs were synthesized following a seeded growth procedure reported elsewhere.^[2]^ Initially, CTAB (3.5 mL, 0.14 M) and HAuCl_4_ (0.12 mL, 15 mM) were mixed, followed by the rapid addition of freshly prepared ice-cold NaBH_4_ (0.50 mL, 10 mM) under vigorous stirring in 20 mL scintillation vial at 26 °C. The resulting brownish-yellow seed solution was stirred for 2 min and subsequently maintained at 26 °C for 0.5 to 2 h to complete Au seed formation. To prepare a growth solution, step, solutions of HAuCl_4_ (0.50 mL, 15 mM), AgNO_3_ (0.40 mL, 4.0 mM), and AA (0.124 mL, 78.8 mM) were sequentially added to the solution of CTAB (8.86 mL, 0.125 M). Subsequently, 0.10 mL of the seed solution was introduced into the growth solution, and the mixture was left undisturbed at 28 °C overnight.

**1.2.3 Depletion-induced fractionation of AuNRs.** Following AuNR synthesis the product contained spherical nanoparticles, which were removed by depletion-induced flocculation reported elsewhere.^[3,4]^ Briefly, a 25 wt% solution of CTAC to a final concentration of 0.05 – 0.3 M was added to the solution of as-synthesized AuNRs. The mixture was left undisturbed in a graduated cylinder for 1-3weeks. The supernatant was then carefully removed, and the precipitated AuNRs were collected and re-dispersed under sonication in a 10 mM CTAC solution. This depletion-induced flocculation step was repeated 2-4 times, with progressive reduction of CTAC concentration.

**1.2.4. Functionalization of AuNRs with thiol-terminated polystyrene.** After depletion-induced flocculation step, the solution of AuNR was concentrated by centrifugation from an initial volume of 1.5 mL to approximately 20 μL, followed by the removal of the supernatant. The concentrated AuNR solution was sonicated for ~5 s and subsequently introduced into 1.5 mL thiol-terminated polystyrene (PS) solution in DMF. The polymer feed concentrations in this mixture were in the range of 0.003-10 mg/mL. After 20-min sonication, the resulting mixture was left undisturbed in a 30 ^o^C water bath overnight. Subsequently to that, the AuNRs were separated *via* 4 cycles of centrifugation (6000 *g*, 10 min, 20 °C), each followed by the supernatant removal, and redispersed in DMF.

## 1.3  **Characterization of AuNRs**

Transmission electron microscopy (TEM) images were obtained using a Hitachi HT7700 microscope at 100 kV. The energy-dispersive X-ray (EDX) element mapping was carried out at 200 kV using a JEM-F200 with the JEOL Field Emission Transmission Electron Microscope. The samples for imaging were prepared by drop-casting a solution of AuNRs onto carbon-coated 300 mesh copper grids and drying it in air at room temperature.

Extinction spectra of AuNRs were acquired using a Cary 5000 UV-Vis-NIR or PerkinElmer Lambda 1050 UV-Vis-NIR spectrophotometers. Proton nuclear magnetic resonance (^1^H-NMR) measurements were carried on a 700 MHz Agilent DD2 NMR spectrometer.

Tomographic tilt series of the samples were acquired in an ADF-STEM mode (collecting angle: 59~200 mrad) using a Thermo Fisher Talos F200X microscope with an acceleration voltage of 200 kV. The samples were imaged over a tilt range of around ± 65° at 2~3° increments with 1024×1024 pixels per image. To mitigate dose-rate–induced damage and scan distortion, four sequential frames were recorded at each tilt angle using a 500 ns dwell time per pixel. Tomography data preprocessing was performed in MATLAB, using center of mass and common line to align the tilt series. Tomographic reconstructions were obtained using the iterative expectation–maximization (EM) algorithm^[5]^. The final reconstructed tomograms were imported into Avizo for visualization.

Polymer grafting density on the AuNR surface was determined using the method reported elsewhere. ^[6]^ Following ligand exchange described in Section 1.2.4, the supernatant was collected and examined by NMR spectroscopy. The grafting density of PS on the AuNR surface was calculated as

$\sigma= \frac{{[PS]}_{\mathrm{tot}}-{[PS]}_{\mathrm{free}}}{\left[ AuNR \right]\times{SA}_{\mathrm{AuNR}}}$ ,

where [*PS*]_tot_ is the total molar concentration of thiol-terminated polystyrene introduced in the solution, [*PS*]_free_ is the molar concentration of the polymer in the supernatant after the ligand exchange, [*AuNR*] is the molar concentration of AuNR in the solution, and *SA*_AuNR_ is the surface area of a single AuNR. The concentrations [*PS*]_tot_ and [*PS*]_free_ were measured by NMR.

All measurements were performed in Norell standard series NMR tubes (5 mm outer diameter, 4.2 mm inner diameter). To ensure the experiment accuracy, all parameters, including acquisition time, relaxation delay and 90-degree pulse, were calibrated to exceed the longest spin-lattice relaxation time (T1) for thiol-terminated PS molecules with number-average molecular weights of 5 300, 25 000, or 50 000g/mol, denoted as PS-5K, PS-25K, and PS-50K, respectively. Overall, 16 to 1024 transient measurements were performed at 25 ^o^C depending on polymer concentration, with a spectral width of 11160.7 Hz, a digital resolution of at least 0.02 Hz/pt, and a minimum signal-to-noise ratio of 360. The solvent DCM signal at 25^o^C (5.32 ppm) was used as chemical shift reference. Each measurement was performed in triplicate. The results were reported as the mean value with corresponding standard deviation.

For quantification, 50 μL of an internal TMB reference solution in DCM-d_2_ at different concentrations (that is, 1, 10, 50, and 200 mM) was used. **Figure S1** shows calibration graphs for the PS-5K, PS-25K, and PS-50K samples. For each calibration, the integral of the PS peak was normalized to the integral of the internal TMB reference peak. The coefficients of determination (*R*^2^) for the calibration graphs obtained using different internal TMB standards were close to a unity, indicating a strong correlation between the measured and standard polymer concentrations. In addition, the slopes of the linear fits were close to 1.0, indicating agreement between the measured and standard polymer concentrations and confirming the accuracy of the quantification method. To determine [*PS*]_tot_ and [*PS*]_free_, the PS peak integral was normalized to that of the internal TMB standard, and the resulting ratio was converted to the corresponding polymer concentration using the appropriate calibration function for the relevant PS molecular weight and concentration range.

**Figure S1**. Calibration curves used for the determination of PS-5K, PS-25K, and PS-50K concentrations using internal TMB standards (50 μL in DCM-d₂) at concentrations of 1 mM (green), 10 mM (blue), 50 mM (purple), and 200 mM (brown). Each measurement was performed in triplicate, and the results are reported as the mean value with corresponding standard deviation.

**Figure S2** shows experimentally determined polymer grafting density, *σ*, plotted *vs*. the polymer feed ratio (the total number of PS molecules introduced into the ligand exchange solution normalized by the total AuNR surface area). The value of *σ* increased with increasing polymer feed ratio and approached a plateau, suggesting surface saturation.

**Figure S2**. Variation in the polymer grafting density on the AuNR surface, plotted as a function of polymer feed ratio. Each measurement was performed in triplicate, and results are reported as the mean value with corresponding standard deviation.

# 2. **Etching of penta-twinned AuNRs functionalized with PS-5K**

**Figure S3** shows that for penta-twinned AuNRs coated with PS-5K at *σ* = 0.22 ± 0.05 chains/nm^2^ and subjected for 15-h etching at varying molar ratio [Fe³⁺]/[Au⁰] (denoted as *R*), significant AuNR defragmentation was observed.

**Figure S3.** TEM images of penta-twinned AuNRs capped with PS-5K at *σ* = 0.22 ± 0.05 chains/nm^2^ and subjected to oxidative etching at varying *R* for 15 h.

# 3. **Etching of single-crystal AuNRs functionalized with PS-5K**

Single-crystal AuNRs coated with PS-5K at *σ* = 0.29 ± 0.11 chains/nm^2^ and subjected for 15-h etching at *R* = 5.0 exhibited the change in the average AuNR length from 52 ± 6 to 47 ± 6 nm, while the average diameter remained unchanged at 17 ± 3 nm (Figure S4a). At *R* =10.0 after 15 h-etching, single-crystal AuNRs exhibited uniform thinning, but maintained a smooth surface (Figure S4b).

**Figure S4**. Etching-induced change in the shape of single-crystal AuNRs capped with PS-5K at $\sigma$ = 0.29 ± 0.11 chains/nm^2^ after 15-h etching at (a) *R* = 5.0, and (b) *R* = 10.0.

# 4. **Analysis of the topography of etched penta-twinned AuNRs**

An average diameter of the AuNRs was determined by analyzing their TEM images using a custom Python-based image analysis (Code available at Github: [ https://github.com/Taylorwutianyi/Automated-Nanorod-Morphology-Analysis]). Individual etched AuNRs were automatically segmented to determine their projected surface area, *A*_proj_, maximum principal axis length, *L*, the average pit-to-pit distance, and the average pit depth.

To establish a geometry-independent averaged descriptor of the rod transverse dimensions, each AuNR was approximated as a rectangle of length *L* and an effective average diameter, *D*_avg_, that was determined as

$D_{\mathrm{avg}}=\frac{A_{\mathrm{proj}}}{L}$,

thus, representing the width of a hypothetical smooth-surface AuNR with a projected area and length identical to the surface-corrugated AuNR. This method provided a parameter for inter-sample comparison, which did not depend on individual corrugation features.

The surface roughness of the AuNRs was characterized by the average pit-to-pit distance and pit depth, both reported in the main text. The pit-to-pit distance was determined as the distance between the centers of two adjacent local maxima (crest-to-crest spacing) along the longitudinal axis of the AuNRs. The pit depth was determined as the difference between the local maximum and the adjacent local minimum, measured perpendicular to the AuNR long axis, as illustrated in **Figure S5**. The values for *D*_avg_, the average pit-to-pit distance and the average pit depth were determined for at least, N ≥ 200 AuNRs for each etching condition.

**Figure S5**. 2D illustration of the determination of average pit-to-pit distance and the average pit depth for the etched penta-twinned AuNR.

# 5. **Extinction spectra of etched AuNRs functionalized with PS-5K**

Etching of AuNR resulted in changes in their ultraviolet–visible–near-Infrared (UV-Vis-NIR) spectra (**Figure S6**). At different etching ratios, *R*, a similar trend was observed, that is, a stronger red shift occurred at a lower polymer *σ*, which was accompanied by spectral broadening and a decreased extinction intensity.

**Figure S6**. Evolution of extinction spectra of AuNRs capped with PS-5K at varying σ and subjected to etching at *R* = 10.0 (a), and *R* = 20.0 (b). The colored solid lines correspond to etched AuNRs end-tethered with PS-5K with *σ* values indicated in the insets. The spectra of AuNRs prior to etching (pristine AuNRs) are shown with dashed red lines.

# 6. **Stability of Etched AuNRs**

To test the long-term stability of the AuNRs, the residual etchant was removed via three cycles of centrifugation, and the AuNRs were dried and stored for 90 days. Upon redispersion of these AuNRs in DMF, their spectra exhibited $\lambda_{\mathrm{LSPR}}$ comparable to the initial etched AuNRs (**Figure S7a**). Furthermore, TEM images of the AuNRs stored for 90 days and redispersed in DMF confirmed no aggregation or structural changes (**Figure S7b**).

**Figure S7**. Long-term stability of etched AuNRs functionalized with PS-5K at $\sigma$ = 0.78 $\pm$ 0.05 chains/nm^2^ (*R* = 10.0 for 15-h etching). (a) UV-Vis-NIR extinction spectra and (b) the corresponding TEM images of the etched AuNRs, acquired immediately after etching and following 90-day storage in the dry state and subsequent redispersion in DMF.

# 7. **Etching of penta-twinned AuNRs functionalized with PS-25K**

**Figure S8** shows representative TEM images and the corresponding analysis of AuNRs functionalized with PS-25K, which were etched at *R =* 5.0 for 15 h. With increasing $\sigma$, a transition occurred from distinct pitting to mild roughening of the AuNRs (Figure S8a). No statistically significant change in the AuNR length was observed (Figure S8b), while the average effective AuNR diameter decreased at lower values $\sigma$ values, due to the formation of surface pits on the long AuNR side (Figure S8c). Figures S8d and e showed that with increasing $\sigma$, the average pit-to-pit distance remained constant, while the average pit depth reduced.

**Figure S8.** Characterization of penta-twinned AuNRs capped with PS-25K and etched at *R* = 5.0 for 15 h. (a) Representative TEM images of etched AuNRs capped with PS-25K at varying $\sigma$. Scale bars are 100 nm. (b) Variation in the average length of etched AuNRs, plotted versus polymer grafting density $\sigma$. (c) Variation in the average diameter of etched AuNRs at varying $\sigma$. The average AuNR length and diameter prior to etching were 92 ± 7 and 27 ± 5 nm, respectively, as shown with dashed horizontal lines in (b) and (c). Average pit-to-pit distance (d) and pit depth (e) of etched AuNRs at varying $\sigma$.

# 8. **Correlation between optical and morphological evolution**

**Figure S9** shows the variation in ${\Delta\lambda}_{\mathrm{LSPR}}$ plotted as a function of the aspect ratio (length/effective diameter) for the etched AuNRs which were functionalized with PS-5K or PS-25K. In this figure, the standard deviation in aspect ratio reflects the distribution in the AuNR length and effective diameter (the latter accounts for the variation in pit depth). The pit-to-pit distance showed no statistically significant difference with changing grafting densities.

**Figure S9.** Variation in ${\Delta\lambda}_{\mathrm{LSPR}}$ with aspect ratio for AuNRs functionalized with PS-5K or PS-25K and etched at *R* = 5.0 for 15 h. The dashed line shows linear regression fit with the corresponding coefficient of determination (R^2^). The horizontal and vertical error bars represent the standard deviations of the aspect ratio and the ${\Delta\lambda}_{\mathrm{LSPR}}$, respectively, derived from three independent experiments.

# 9. **Etching of penta-twinned AuNRs functionalized with PS-50K**

Functionalization of penta-twinned AuNRs with PS-50K resulted in maximum *σ* = 0.07 ± 0.02 chains/nm^2^ achieved at the feed ratio of 919 chains/nm^2^. Further increase in the polymer feed ratio did not change the value of *σ*. This effect arose from the steric crowding of the high-molecular-weight polymer chains on the AuNR surface. At *R* = 5.0 and etching time of 15-h (the etching conditions used for the AuNRs capped with PS-5K and PS-25K), the AuNRs functionalized with PS-50K at *σ* = 0.07 ± 0.02 chains/nm^2^ transformed into nanospheres (**Figure S10**). Such transformation suggested insufficient surface passivation against oxidative etching at low grafting density of PS-50K.

**Figure S10**. Transformation of AuNRs end-capped with PS-50K into nanospheres after 15-h etching at *R* = 5.0.

# 10. **Etching of AuNRs functionalized with alternative thiol-terminated ligands**

To decouple the impact of the polymer backbone from the thiol anchoring group in oxidative etching, control experiments were performed using alternative thiol-terminated ligands. First, AuNRs were functionalized with 1-dodecanthiol (DDT) with a molecular weight of 202.4 g/mol. The ligand exchange procedure was carried out as described in Section 1.2.4, with the incubation time of 3.5-h and the concentration of DDT in the solution in the range of 0.2-2000 ng/mL (corresponding to 5.95$\times$10^11^ – 5.95$\times$10^14^ molecules/mL and the feed ratio in the range from 0.003 to 3.0 molecules/nm^2^, respectively). Notably, the concentration of DDT of ~1 µM (6$\times$10^14^ molecules/mL) was reported for the preparation of dense self-assembled DDT monolayers on gold surface,^[7]^ while lower DDT feed ratios were deliberately chosen to provide grafting densities close to those of PS molecules.

At the feed ratio of 0.003, 0.03 and 0.3 molecules/nm^2^, upon etching, the DDT-functionalized AuNRs transformed into smooth-surface rice-shaped nanoparticles (**Figure S11a**), in striking difference with the results of etching of the AuNRs end-capped with PS-5K or PS-25K. For the AuNRs functionalized with DDT at the highest feed ratio of 3.0 molecules/nm^2^, that is, close to the concentration leading to the formation of the dense DDT monolayer ^[7]^, etching was largely suppressed.

To evaluate the role of the nature of the polymer ligands, preliminary experiments were performed for the AuNRs functionalized with a thiol-terminated poly(methyl methacrylate) (PMMA) with a molecular weight of 6,500 g/mol. The ligand exchange procedure is described in Section 1.2.4, and polymer feed ratio was 0.56 molecules/nm^2^. Following 15-h etching at *R* = 5.0 (similar to the etching conditions used for the AuNRs end-capped with PS-5K), the PMMA-functionalized AuNRs exhibited random and shallow surface corrugations (**Figure S11b**), in comparison with those observed for the etched PS-functionalized AuNRs. We note that it is challenging to find a non-aromatic polymer counterpart that perfectly matches the PS brush thickness and grafting density used in the present study, and thus this comparison is qualitative. Nevertheless, these results indicate that the polymer nature plays a role in the resulting morphology of etched AuNRs, which would be a subject of future studies.

**Figure S11**. Morphology of AuNRs functionalized with alternative thiol-terminated ligands, following oxidative etching. (a) Representative TEM images of AuNRs functionalized with DDT at feed ratios of (*left to right*) 0.003, 0.03, 0.3 and 3.0 molecules/nm² (b) PMMA at feed ratio of 0.56 molecules/nm^2^. Etching was conducted at *R* = 10.0 for 15 h.

# 11. **Calculation of thickness of the polymer layer on the AuNR surface**

The average spacing between end-grafted polymer chains on the AuNR surface was calculated as

$d= \frac{1}{\sqrt{\sigma}}$,

where the polymer grafting density $\text{σ}$ in chains/nm² was assumed to be uniform on the AuNR surface).

For PS-5K and PS-25K with grafting density in the range of 0.63-0.85 chains/nm^2^ the intermolecular spacing was in the range of 1.07-1.34 nm (Table S1). The radius of gyration of the polymer molecules in good solvent, such as DMF, was estimated using the relation^[8]^

$R_{g}= aN^{3/5}$,

where $a$ is the effective monomer size ($a$ ≈ 0.19 nm for polystyrene ^[9]^)and *N* is the degree of polymerization of the polymer.

For PS-5K and PS-25K with degrees of polymerization of 51 and 240, respectively, the value of *R*_g_ was calculated to be 2.0 and 5.1 nm, respectively (Table S1). Because the calculated *R*_g_ values were substantially larger than the interchain spacing on the AuNR surface, the thickness of the polymer layer on the surface of AuNRs in good solvent was estimated using the Alexander–de Gennes relationship ^[8]^as

$$h=N\sigma^{1/3}a^{5/3}$$

Table S1 shows that in DMF (the calculated polymer brush thickness on the surface of AuNRs was approximately five times greater for PS-25K than for PS-5K.

**Table S1.** Characteristics of polystyrene molecules and estimated polymer layer thickness

| **Data point in Figure 3 (main text)** | **M_n_**  **(g/mol)** | ***N*** | ***R*_g_**  **(nm)** | ***σ***  **(chains/nm^2^)** | ***d***  **(nm)** | $\boldsymbol{h}$  **(nm)** |
| --- | --- | --- | --- | --- | --- | --- |
| A | 5,300 | 51 | 2.0 | 0.63 | 1.26 | 2.7 |
| B | 5,300 |  |  | 0.78 | 1.13 | 2.9 |
| C | 25,000 | 240 | 5.1 | 0.64 | 1.25 | 13.0 |
| D | 25,000 |  |  | 0.85 | 1.08 | 14.3 |

We note that the calculated polymer layer thicknesses correspond to the average equilibrium value in a good solvent and are expected to exceed the apparent thickness observed in TEM images due to polymer collapse, chain interpenetration, and drying effects during sample preparation.

# 12. **Energy-dispersive X-ray elemental mapping of etched AuNRs**

The energy-dispersive X-ray (EDX) elemental mapping was used to confirm the presence of iron on the surface of etched AuNRs. **Figure S12** shows high-angle annular dark-field (HAADF)-STEM image and the corresponding Au and Fe STEM-EDX maps of the etched AuNR, with iron distributed across the AuNR surface. We note that previous studies reported weak coordination between Fe³⁺ ions and electron-rich aromatic rings of PS via cation–*π* interactions. ^[10,11]^

**Figure S12.** HAADF-STEM image of etched AuNRs (left) and corresponding STEM-EDX elemental maps of Au (middle) and Fe (right). All scale bars are 30 nm.

# 13. **Simulations**

## 13.1 **Finite-difference time-domain simulations**

In Finite-Difference Time-Domain (FDTD) simulations, the models of etched AuNR were fabricated using TEM images of AuNRs. The PS-5K–functionalized AuNRs had average length of 81 nm, a maximum width of 19 nm, and an average pit depth of 3 nm, whereas the PS-25K–functionalized AuNRs had the average length of 85 nm, a maximum diameter of 21 nm, and a pit depth of 2 nm. The mesh size of 0.50 nm was applied across the simulation volume. The planar polarized wave source was chosen to simulate the extinction spectra in the spectral range of 450-1300 nm. For the bare AuNR models, the surrounding environment was assigned the refractive index of DMF (n = 1.43). For the polymer-coated models, the effective refractive index ($n_{\mathrm{eff}}$) of the polymer layer was calculated as follows.

Because DMF is a good solvent for PS ^[12]^, and the polymer brush assumes a swollen random coil conformation, the value of $n_{\mathrm{eff}}$ was determined by calculating the volume fraction, $\emptyset_{\mathrm{PS}}$, of PS within the swollen PS/DMF mixture. A smooth-surface capsule model possessing a projected area and length identical to the surface-corrugated AuNR was utilized for geometric baseline calculations, as indicated in Section 4. The length and effective diameter (*r*) of bare AuNRs were used to determine the bare core volume ($V_{bare AuNR}$). The swollen shell volume ($V_{swollen PS}$) was determined by subtracting the bare core volume from the total volume of the AuNRs coated with a swollen polymer ($V_{swollen PS-AuNR}$), where the thickness ($h_{swollen PS}$) was derived using theoretical polymer brush models in a good solvent (Table S1). The dry polymer layer volume ($V_{dry PS}$) was determined by using the dry layer thickness ($h_{dry PS}$) measured experimentally from the TEM images. These structural parameters are summarized in **Table S2**.

**Table S2**. Structural parameters of bare and polymer-coated etched AuNRs

| Sample | Length  (nm) | *r*  (nm) | $V_{bare AuNR}$  (nm^3^) | $h_{swollen PS}$  (nm) | $V_{swollen PS-AuNR}$  (nm^3^) | $V_{swollen PS}$  (nm^3^) | $h_{dry PS}$  (nm) | $V_{dry PS-AuNR}$  (nm^3^) | $V_{dry PS}$  (nm^3^) |
| --- | --- | --- | --- | --- | --- | --- | --- | --- | --- |
| PS-5K (A) | 81.2 | 15.4 | 14169 | 2.7 | 27070 | 12901 | 2.4 | 25403 | 11234 |
| PS-25K (C) | 84.9 | 18.5 | 21163 | 13.0 | 149411 | 128247 | 7.6 | 79266 | 58102 |

The volume fraction of the polymer within a solvated layer was determined as

$$\emptyset_{\mathrm{PS}}=\frac{V_{dry PS}}{V_{swollen PS}}$$

The calculation yielded $\emptyset_{\mathrm{PS}}$ = 0.871 for the PS-5K layer. Conversely, the PS-25K with $\emptyset_{\mathrm{PS}}$ = 0.453 was highly swollen with DMF. The calculations yielded an effective refractive index of $n_{\mathrm{eff}}$ = 1.55 for the PS-5K brush layer and $n_{\mathrm{eff}}$= 1.49 for the PS-25K brush layer. Importantly, these calculations rely on an ideal smooth-surface model. Etched AuNRs possess a corrugated surface, and hence a significantly larger surface area. Thus, the actual polymer density near the gold interface may be lower.

Using the calculated volume fractions, the value of $n_{\mathrm{eff}}$ for the solvated polymer layer was determined via the FDTD Lorentz-Lorenz effective medium approximation ^[13]^, using the refractive indices of PS ($n_{\mathrm{PS}}$ = 1.57) ^[14]^ and DMF ($n_{\mathrm{DMF}}$ = 1.43). All simulated extinction spectra were obtained by averaging the results from six linear polarization excitations (consisting of two orthogonal polarizations for each of the three propagation directions along the x-, y-, and z-axis) to accurately model unpolarized experimental conditions.

$$\frac{n_{\mathrm{eff}}^{2}-1}{n_{\mathrm{eff}}^{2}+2}=\phi_{\mathrm{PS}}\frac{n_{\mathrm{PS}}^{2}-1}{n_{\mathrm{PS}}^{2}+2}+\left( 1-\phi_{\mathrm{PS}} \right)\frac{n_{\mathrm{DMF}}^{2}-1}{n_{\mathrm{DMF}}^{2}+2}$$

## 13.2 **Density functional theory simulations**

The adsorption behavior of the polymer species and its influence on Au surface etching were simulated using Density Functional Theory (DFT). The crystallographic surface, Au{100}, was simulated for the long sides of penta-twinned AuNRs. Each surface was constructed as a 4 × 4 periodic slab model containing 64 Au atoms. The DFT calculations were carried out using the Vienna *ab initio* Simulation Package ^[15,16]^ within a plane-wave framework ^[17]^. Exchange–correlation effects were treated using the spin-polarized generalized gradient approximation in the Perdew–Burke–Ernzerhof form ^[17]^, while core–valence interactions were described using the projector augmented wave method. ^[18,19]^ A plane-wave kinetic energy cutoff of 450 eV was utilized. Dispersion interactions were incorporated using Grimme’s DFT-D3 scheme with zero damping ^[20]^ to account for long-range van der Waals forces. To prevent spurious interactions between periodic slabs, a vacuum layer of 15 Å was introduced along the direction perpendicular to the surface.

# **References**

[1]	A. Sánchez-Iglesias, N. Winckelmans, T. Altantzis, S. Bals, M. Grzelczak, L. M. Liz-Marzán, “High-yield seeded growth of monodisperse pentatwinned gold nanoparticles through thermally induced seed twinning” *Journal of the American Chemical Society* **139**, **(**2017**)**, 107–110.

[2]	B. Nikoobakht, M. A. El-Sayed, “Preparation and growth mechanism of gold nanorods (NRs) using seed-mediated growth method” *Chemistry of Materials* **15**, **(**2003**)**, 1957–1962.

[3]	Z. Cheng, M. R. Jones, “Separation of nanoparticle seed pseudoisomers via amplification of their crystallographic differences” *Journal of the American Chemical Society* **145**, **(**2023**)**, 27702–27707.

[4]	M. Mayer, L. Scarabelli, K. March, T. Altantzis, M. Tebbe, M. Kociak, S. Bals, F. J. Garciá De Abajo, A. Fery, L. M. Liz-Marzán, “Controlled living nanowire growth: precise control over the morphology and optical properties of AgAuAg bimetallic nanowires” *Nano Letters* **15**, **(**2015**)**, 5427–5437.

[5]	Todd. K. Moon, “The Expectatio Maximization Algorithm” *IEEE Signal Processing Magazine* **(**1996**)**, 47–60.

[6]	J. Kang, Y. X. Wang, F. Peng, N. N. Zhang, Y. Xue, Y. Yang, E. Kumacheva, K. Liu, “Oxidative elimination and reductive addition of thiol-terminated polymer ligands to metal nanoparticles” *Angewandte Chemie - International Edition* **61**, **(**2022**)**, e202202405.

[7]	J. C. Love, L. A. Estroff, J. K. Kriebel, R. G. Nuzzo, G. M. Whitesides, “Self-assembled monolayers of thiolates on metals as a form of nanotechnology” *Chemical Reviews* **105**, **(**2005**)**, 1103–1169.

[8]	M. Rubinstein, R. H. Colby, *Polymer Physics*, Oxford University Press: Oxford, **(**2003**)**.

[9]	L. J. Fetters, N. Hadjichristidis, J. S. Lindner, J. W. Mays, “Molecular Weight Dependence of Hydrodynamic and Thermodynamic Properties for Well-Defined Linear Polymers in Solution” *Journal of Physical and Chemical Reference Data* **23**, **(**1994**)**, 619–640.

[10]	D. R. Evans, N. L. P. Fackler, Z. Xie, C. E. F. Rickard, P. D. W. Boyd, C. A. Reed, “π-Arene/cation structure and bonding. Solvation versus ligand binding in iron(III) tetraphenylporphyrin complexes of benzene, toluene, p-xylene, and [60]fullerene” *Journal of the American Chemical Society* **121**, **(**1999**)**, 8466–8474.

[11]	Ç. A. Demircan, U. Bozkaya, “Transition Metal Cation-π Interactions: Complexes Formed by Fe2+, Co2+, Ni2+, Cu2+, and Zn2+ Binding with Benzene Molecules” *Journal of Physical Chemistry A* **121**, **(**2017**)**, 6500–6509.

[12]	Z. Nie, D. Fava, M. Rubinstein, E. Kumacheva, “‘Supramolecular’ assembly of gold nanorods end-terminated with polymer ‘pom-poms’: Effect of pom-pom structure on the association modes” *Journal of the American Chemical Society* **130**, **(**2008**)**, 3683–3689.

[13]	F. Pretorius, W. W. Focke, R. Androsch, E. du Toit, “Estimating binary liquid composition from density and refractive index measurements: A comprehensive review of mixing rules” *Journal of Molecular Liquids* **332**, **(**2021**)**, 115893.

[14]	S. N. Kasarova, N. G. Sultanova, C. D. Ivanov, I. D. Nikolov, “Analysis of the dispersion of optical plastic materials” *Optical Materials* **29**, **(**2007**)**, 1481–1490.

[15]	G. Kresse, “Ab initio molecular dynamics for liquid metals” *Journal of Non-Crystalline Solids 192&193* **(**1995**)**, 222–229.

[16]	G. Kresse, J. Furthmiiller, “Efficiency of ab-initio total energy calculations for metals and semiconductors using a plane-wave basis set” *Computational Materials Science* **6**, **(**1996**)**, 15–50.

[17]	M. C. Payne, M. P. Teter, D. C. Allan, T. A. Arias, J. D. Joannopoulos, “Iterative minimization techniques for ab initio total-energy calculations: molecular dynamics and conjugate gradients” *Reviews of Modern Physics* **64**, **(**1992**)**, 1045–1097.

[18]	G. Kresse, D. Joubert, “From ultrasoft pseudopotentials to the projector augmented-wave method” *Physical Review B* **59**, **(**1999**)**, 1758–1775.

[19]	P. E. Blochl, “Projector augmented-wave method” *Physical Review B* **50**, **(**1994**)**, 17953–17979.

[20]	S. Grimme, J. Antony, S. Ehrlich, H. Krieg, “A consistent and accurate ab initio parametrization of density functional dispersion correction (DFT-D) for the 94 elements H-Pu” *Journal of Chemical Physics* **132**, **(**2010**)**, 154104.
